# Establishment of a nomogram with EMP3 for predicting clinical outcomes in patients with glioma: A bi‐center study

**DOI:** 10.1111/cns.13701

**Published:** 2021-07-16

**Authors:** Anke Zhang, Houshi Xu, Zeyu Zhang, Yibo Liu, Xiaying Han, Ling Yuan, Yunjia Ni, Shiqi Gao, Yuanzhi Xu, Sheng Chen, Junkun Jiang, Yike Chen, Xiaotao Zhang, Meiqing Lou, Jianmin Zhang

**Affiliations:** ^1^ Department of Neurosurgery School of Medicine The Second Affiliated Hospital Zhejiang University Hangzhou China; ^2^ Department of Neurosurgery School of Medicine Shanghai General Hospital Shanghai Jiao Tong University Shanghai China; ^3^ Department of Orthopedics School of Medicine Shanghai General Hospital Shanghai Jiao Tong University Shanghai China; ^4^ Tongji University Shanghai China

**Keywords:** biomarker, glioma, immune infiltration, nomogram, prognosis

## Abstract

**Aim:**

To demonstrate the clinical value of epithelial membrane protein 3 (EMP3) with bioinformatic analysis and clinical data, and then to establish a practical nomogram predictive model with bicenter validation.

**Methods:**

The data from CGGA and TCGA database were used to analyze the expression of EMP3 and its correlation with clinical prognosis. Then, we analyzed EMP3 expression in samples from 179 glioma patients from 2013 to 2017. Univariate and multivariate cox regression were used to predict the prognosis with multiple factors. Finally, a nomogram to predict poor outcomes was formulated. The accuracy and discrimination of nomograms were determined with ROC curve and calibration curve in training and validation cohorts.

**Results:**

EMP3 was significantly higher in higher‐grade glioma and predicted poor prognosis. In multivariate analysis, high expression of EMP3 (HR = 2.842, 95% CI 1.984–4.071), WHO grade (HR = 1.991, 95% CI 1.235–3.212), and IDH1 mutant (HR = 0.503, 95% CI 0.344–0.737) were included. The nomogram was constructed based on the above features, which represented great predictive value in clinical outcomes.

**Conclusion:**

This study demonstrated EMP3 as a novel predictor for clinical progression and clinical outcomes in glioma. Moreover, the nomogram with EMP3 expression represented a practical approach to provide individualized risk assessment for glioma patients.

## Graphical abstract

The data from CGGA and TCGA database were used to analyze the expression of EMP3 and its correlation with clinical prognosis and immune infiltration. Then, we analyzed EMP3 expression by IHC staining in samples from 179 glioma patients from 2013 to 2017. Univariate and multivariate cox regression were used to predict the prognosis with multiple factors. Finally, a nomogram to predict poor outcomes was formulated. The accuracy and discrimination of nomograms were determined with ROC curve and calibration curve in training and validation cohorts.

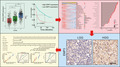

## 1 INTRODUCTION

Glioma is the most common malignant tumor of the central nervous system, with a 5‐year overall survival (OS) rate of approximately 36%, and accounts for more than 70% of intracranial malignant tumors.^1^, ^2^, ^3^ Regardless of tumor malignancy and aggressive treatment, the average median OS time is still only 12–18 months.^4^, ^5^ Although various therapeutic modalities are available, such as surgical resection, chemotherapy, radiotherapy, and immunotherapy, patients with glioma have low survival rates.

Therapeutic effects are affected by tumor heterogeneity and genetic and epigenetic factors. Moreover, aggressive therapies compromise the quality of life of patients and confer harmful adverse reactions. Therefore, knowledge of mechanisms underlying tumor progression is critical for management strategies and prognostic predictions.

With the development of high‐throughput microarray technology, gene expression profiling has been widely used to determine signatures or biomarkers associated with tumor progression and clinical prognosis.^6^, ^7^, ^8^ Although many studies of the prognostic biomarkers have been reported, few have been validated in the clinical setting. Genetic signatures identified from four different published microarray datasets have been validated in glioma cohorts.^9^, ^10^, ^11^ However, the clinical value of these genetic signatures is undetermined and has not been applied in clinical practice.

The tumor immune microenvironment participates in oncogenesis and tumor progression, and affects clinical prognosis.^12^ Several studies have demonstrated the correlation between the tumor immune microenvironment and N6‐methyladenosine (m6A) modification; however, the potential role of m6A modification in immune infiltration is still unclear, especially in glioma. m6A is the most common mRNA modification in diverse cells and has various regulatory functions in tumorigenesis, progression, and immune modulation.^13^ In our current study, we built a model with integrated m6A and immune infiltration data to improve the overall prediction of outcome for glioma patients without using a large number of samples to verify its clinical practicality.^14^ To the best of our knowledge, few studies have applied human glioma samples to verify signatures or biomarkers screened from public datasets. Therefore, we aimed to combine bioinformatic analysis with clinical data to confirm the clinical value of the signature and established a nomogram predictive model with bicenter validation.

EMP3 belongs to the peripheral myelin protein 22 (PMP22)/claudin superfamily of proteins and has four transmembrane domains.^15^, ^16^ Aberrant expression of EMP3 has been found in many cancers. Recently, many studies have focused on the role of EMP3 in tumor progression and malignant transformation.

It has been reported that EMP3 promotes tumor growth and metastasis via the PI3K/AKT pathway, which is highly expressed in upper urinary tract urothelial cancer (UTUC) and hepatocellular carcinoma (HCC).^17^, ^18^ However, several studies considered EMP3 to be a tumor suppressor gene in several solid cancers, including low‐grade glioma (LGG), esophageal carcinoma, and lung cancer.^19^, ^20^, ^21^ One recent study demonstrated that EMP3 has oncogenic properties in high‐grade glioma (HGG), and its overexpression might also predict poor clinical prognosis in primary glioblastoma multiforme (GBM).^22^, ^23^

Given the limited evidence that EMP3 may be a biomarker to predict the prognosis in gliomas, our study was performed to determine the relationship between EMP3 and immune infiltration and clinical outcomes and to establish a practical nomogram predictive model to guide the clinical therapy and assess prognosis.

## 2 MATERIALS AND METHODS

### 2.1 Collection of human‐derived tumor samples

Sample collection and data analysis were approved by Institutional board of the Second Hospital affiliated to Zhejiang University and Shanghai General Hospital affiliated to Shanghai Jiao Tong University. Tumor tissues were recruited between January 2013 and September 2017 from the department of neurosurgery in both of the Second Hospital affiliated to Zhejiang University and Shanghai General Hospital affiliated to Shanghai Jiao Tong University. Inclusion criteria include the following: (1) all glioma patients confirmed with pathohistological diagnosis and immunohistochemistrical staining results for protein expression (EMP3, Ki67, and PHH3), mutant status (P53, IDH1, and ATRXA), and methylation status of MGMT; and (2) patients with glioma is surgically removed for the first time. Exclusion criteria include the following: (1) patients who receive preoperative radiotherapy or chemotherapy; (2) patients with glioma whose histological grading or staining results are uncertain; and (3) patients with no integrated data of 3‐year follow‐up. A total of 179 cases were eligible for inclusion.

### 2.2 Data source and expression analysis

In this study, we analyzed both HGG and LGG. All glioma data sets were obtained from Gliovis (http://gliovis.bioinfo.cnio.es/), including three datasets (CGGA, TCGA, Rembrandt) containing 1948 samples: grade I patients (*n* = 2), grade II patients (*n* = 615), grade III patients (*n* = 663), and grade IV patients (*n* = 668).

### 2.3 Immune cells and bioinformatic analysis

The single sample gene set enrichment analysis (ssGSEA) was used to define an enrichment fraction that represents the absolute enrichment of genomes in each sample with R package “*GSVA*” within a given dataset. Normalized enrichment fractions can be calculated for each type of immune cell. Genome set signature of 28 immune cells were obtained from a previous study.^24^ Gene Set Variation Analysis from R package GSVA was performed to obtain the immune profile of the glioma samples.

### 2.4 Immunohistochemical analysis

Human‐derived glioma samples were fixed in 4% paraformaldehyde at room temperature for 24 h and then embedded in paraffin. The blocks were cut into 8 μm slices for the following analysis. Sections were blocked with 5% goat serum at room temperature for 1 h and then were stained with EMP3 (1:100, Santa Cruz, sc‐81797, USA). After washing with PBS, the sections were incubated with secondary antibody (1:5000, Beijing Zhongshan‐Golden Bridge Technology Co., Ltd) at 37°C for 30 min. The ABC method (Vector Laboratories) was used at room temperature. The images were observed using an AX‐80 microscope (Olympus, Tokyo, Japan) and were analyzed with ImageJ software.

### 2.5 Real‐time PCR

Total RNA was extracted from human glioma core region and adjacent tissues using TRIzol reagent (Invitrogen, Carlsbad, CA, USA). Reverse transcription was carried out by using FastQuant RT kit (Tiangen, Shanghai, China). Real‐time PCR was carried out using SuperReal SYBR Green kit (Tiangen, Shanghai, China) and LightCycler 96 (Roche, Penzberg, Germany). The primer sequences were listed as follow: EMP3 forward: GCCATGTCACTCCTCTTGCT; EMP3 reverse: CTGACATTACTGCAGGCCCA; ACTB forward: CTCACCATGGATGATGATATCGC; ACTB reverse: CCACATAGGAATCCTTCTGACC.

### 2.6 Nomogram construction and validation

The patients from the Second Hospital affiliated to Zhejiang University were considered as training cohorts, and the patients from Shanghai General Hospital were included in validation cohorts. In the survival analysis, the association between traits and overall survival was assessed using Cox regression model. The Kaplan‐Meier survival curves were plotted and compared by log‐rank tests with R packages “survival” and “survminer”. In the training cohort, independent predictive risk factors were screened by multivariate Cox regression analysis. Based on independent factors, the nomogram model was established with the R software version 4.0.1 (https://www.r‐project.org) to predict outcomes in 1‐, 2‐, and 3‐year follow‐ups. The performance evaluation of the nomogram includes c‐index, calibration curves, and ROC curve, which were verified by the calibration curve generated by the validation cohort. ROC curves, sensitivity, and specificity values were generated using R package “pROC”.

### 2.7 Statistical analysis

All statistical analysese were performed using R software 4.0.1, SPSS analysis tools (IBM Corp.), or the Prism8 software program (GraphPad Software). Continuous variables were reported as Mean ± SD. For the clinical data, Student's *t*‐test was used for continuous variables and chi‐square or Fisher's exact test for categorical variables. All tests were two‐sided. Spearman correlation analysis was used for correlation analysis. Both univariate and multivariable Cox regression were performed to estimate clinical outcomes. We used a multivariable model with a forward stepwise regression procedure to screen out the potential predictors that have been reported or assumed to be predictive of poor outcome. Based on the multivariable analysis, we used modeling nomograms to predict prognosis at 1‐, 2‐, and 3‐year follow‐ups. R package “meta” was used for Meta‐analysis. *p* < 0.05 was considered significant for all statistical analyses.

## 3 RESULTS

### 3.1 Increased EMP3 expression predicts poor prognosis in gliomas patients

**FIGURE 1:**
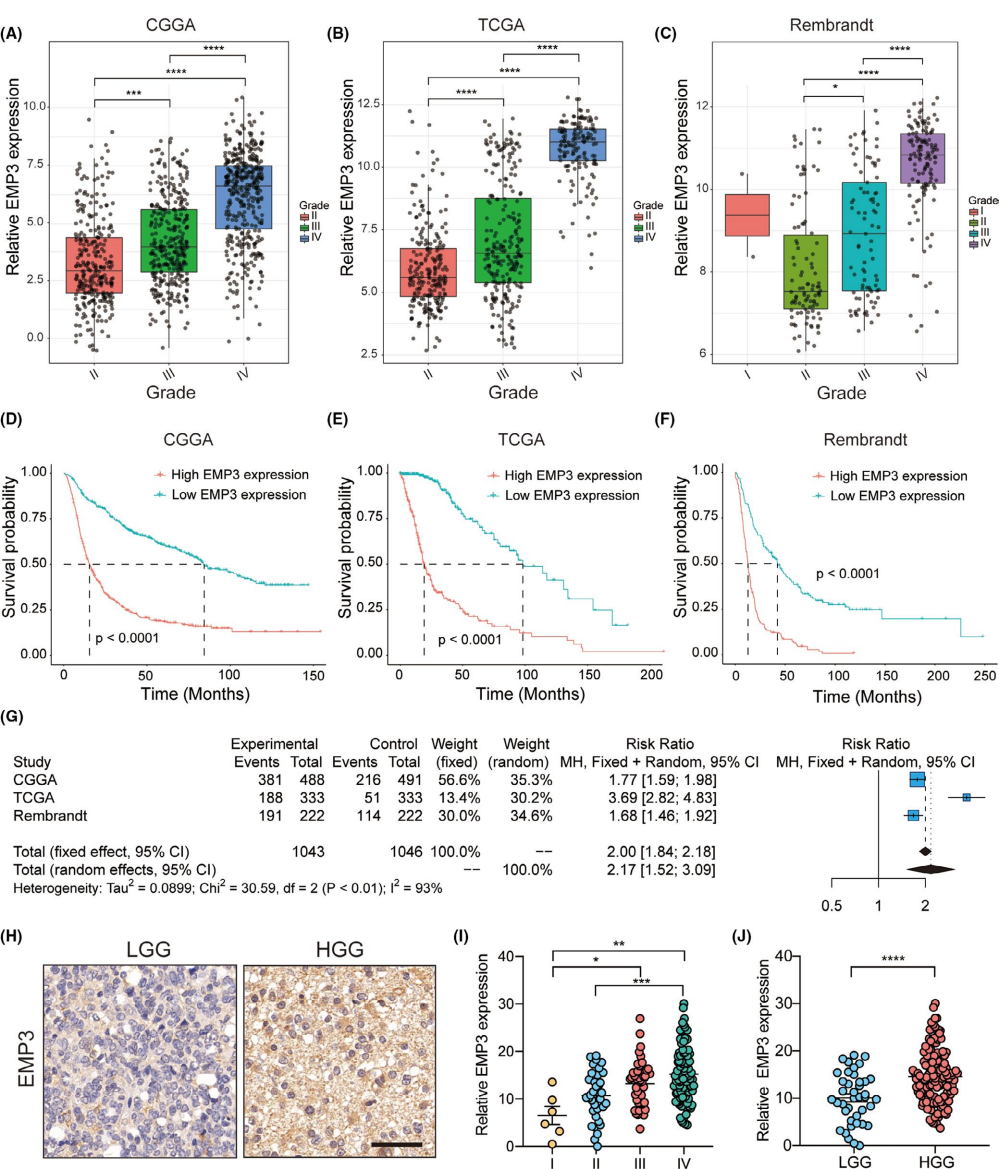
Increased EMP3 predicts progression and poor prognosis in gliomas. (A‐C) The x‐axis represents the WHO grade while the y‐axis represents EMP3 expression value (log2). Based on Wilcoxon test. (A) CGGA, (B) TCGA and (C) Rembrandt. (D‐F) Kaplan‐Meier plots of EMP3 in a variety glioma datasets. (D) CGGA, (E) TCGA, and (F) Rembrandt. (G) Forest plot of the RRs for patients with high EMP3 expression compared with patients with low EMP3 expression. (H‐J) Representation of IHC images and quantification of EMP3 expression in low‐grade glioma and high‐grade glioma. ^*^ *p* < 0.05, ^**^ *p* < 0.01, ^***^ *p* < 0.001, ^****^ *p* < 0.0001

In the initial study, we focused on determining the mechanistic role and clinical value of EMP3 in glioma. To investigate the expression of EMP3 in gliomas at different stages, we analyzed EMP3 mRNA expression in 3 datasets. We observed that the expression of EMP3 was elevated in glioblastoma. In the CGGA dataset, WHO grade III (*n* = 334) and grade IV (*n* = 388) tumors had a significantly higher expression of EMP3 than grade II (*n* = 291) (Figure 1A) tumors. In the TCGA‐GBMLGG dataset, a significant increase in EMP3 expression was confirmed in grade III (*n* = 244) and IV (*n* = 150) tumors (Figure 1B). Furthermore, an upward trend was also observed in the Rembrandt dataset (Figure 1C).

After elucidating the correlation between EMP3 expression and tumor malignancy, we aimed to demonstrate the prognostic value of EMP3. According to the median value of EMP3 expression, patients were divided into high and low EMP3 expression groups. The Kaplan‐Meier curve and survival comparison analysis showed that patients with high EMP3 levels from the CGGA (HR = 1.77, 95% CI = 1.59–1.98), TCGA (HR = 3.69, 95% CI = 2.82–4.83), and Rembrandt (HR = 1.68, 95% CI = 1.46–1.92) datasets had worse overall survival (OS) rates than those with low EMP3 levels (Figure 1D‐F). To improve the stability of this result, we used a fixed‐effects model to summarize the HRs of these three cohorts. The results confirmed that patients with high EMP3 expression had significantly shorter OS times than patients with low EMP3 levels (RR = 2.00, 95% CI = 1.84–2.18, Figure 1G).

To further validate these results, IHC for EMP3 was performed to evaluate EMP3 expression in patient‐derived glioma tissues from two institutions. As expected, there was a significant increase in EMP3 in high‐grade glioma (HGG) compared with low‐grade glioma (LGG) (Figure 1H‐J). In addition, as expected, in comparison with adjacent tissues, a significant increase in EMP3 was revealed in the tumor core region (Figure S1). According to the above data, the expression of EMP3 increased with the progression of glioma, suggesting that EMP3 may be involved in the development of tumor malignancy.

### 3.2 EMP3 regulates immune infiltration and immune activation in gliomas

**FIGURE 2:**
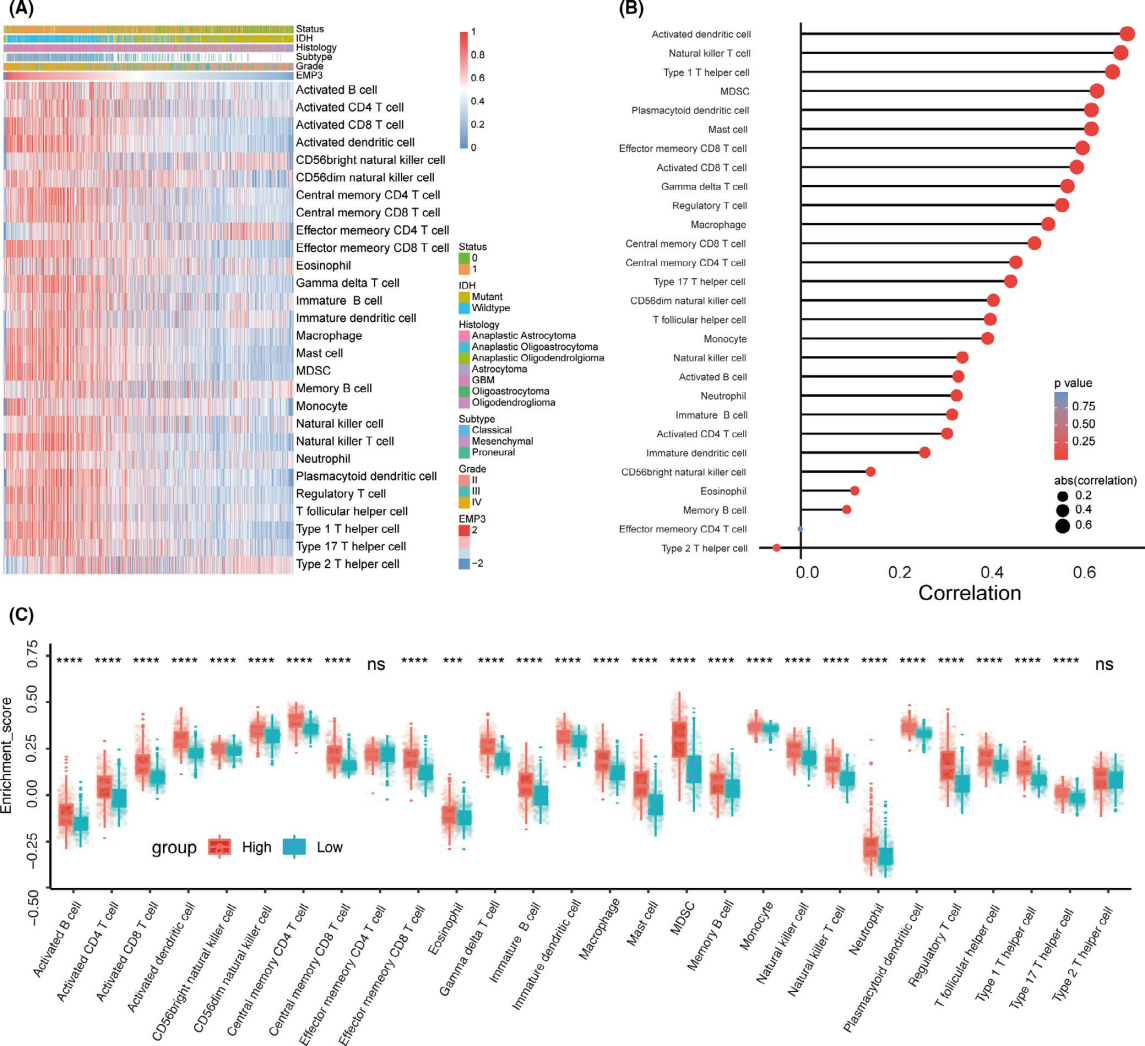
EMP3 is associated with immune infiltration and immune activation in gliomas. (A) Heatmap showing EMP3‐associated relative abundance of 28 immune cells in gliomas, annotations show corresponding clinical features of each sample. (B) The correlation between the ssGSEA scores of 28 immune cells and the expression of EMP3 in gliomas. (C) The fraction of 28 immune cells in EMP3 high and low subgroups. Within each group, the scattered dots represent immune cells ssGSEA values. The thick line represents the median value. The bottom and top of the boxes are the 25th and 75th percentiles (interquartile range, IQR). ^***^ *p* < 0.001, ^****^ *p* < 0.0001

Infiltration of immune cells plays a critical role in a variety of cancers, which may lead to different clinical outcomes. The correlation between the immune profile and prognosis has been reported in several cancers, especially gliomas. The specific presence of EMP3 in lymphoid tissues, including the spleen and thymus, is thought to be directly or indirectly involved in immune system regulation.^25^ However, the role of EMP3 in the tumor immune microenvironment remains unclear. Therefore, we aimed to explore the correlation between EMP3 levels and the status of immune infiltration to reveal the underlying mechanism in affecting the prognosis of gliomas. Twenty‐eight types of immune cells were systematically estimated from the CGGA dataset using the ssGSEA algorithm (Figure 2A). The Spearman method was used to evaluate the relationship between EMP3 expression and immune cell infiltration, which revealed a close correlation between EMP3 expression and the infiltration of T cells, myeloid‐derived suppressor cells (MDSCs), and macrophages (Figure 2B). Notably, the proportions of tumor‐infiltrating immune cells showed significant differences between the high and low EMP3 subsets (Figure 2C).

**FIGURE 3:**
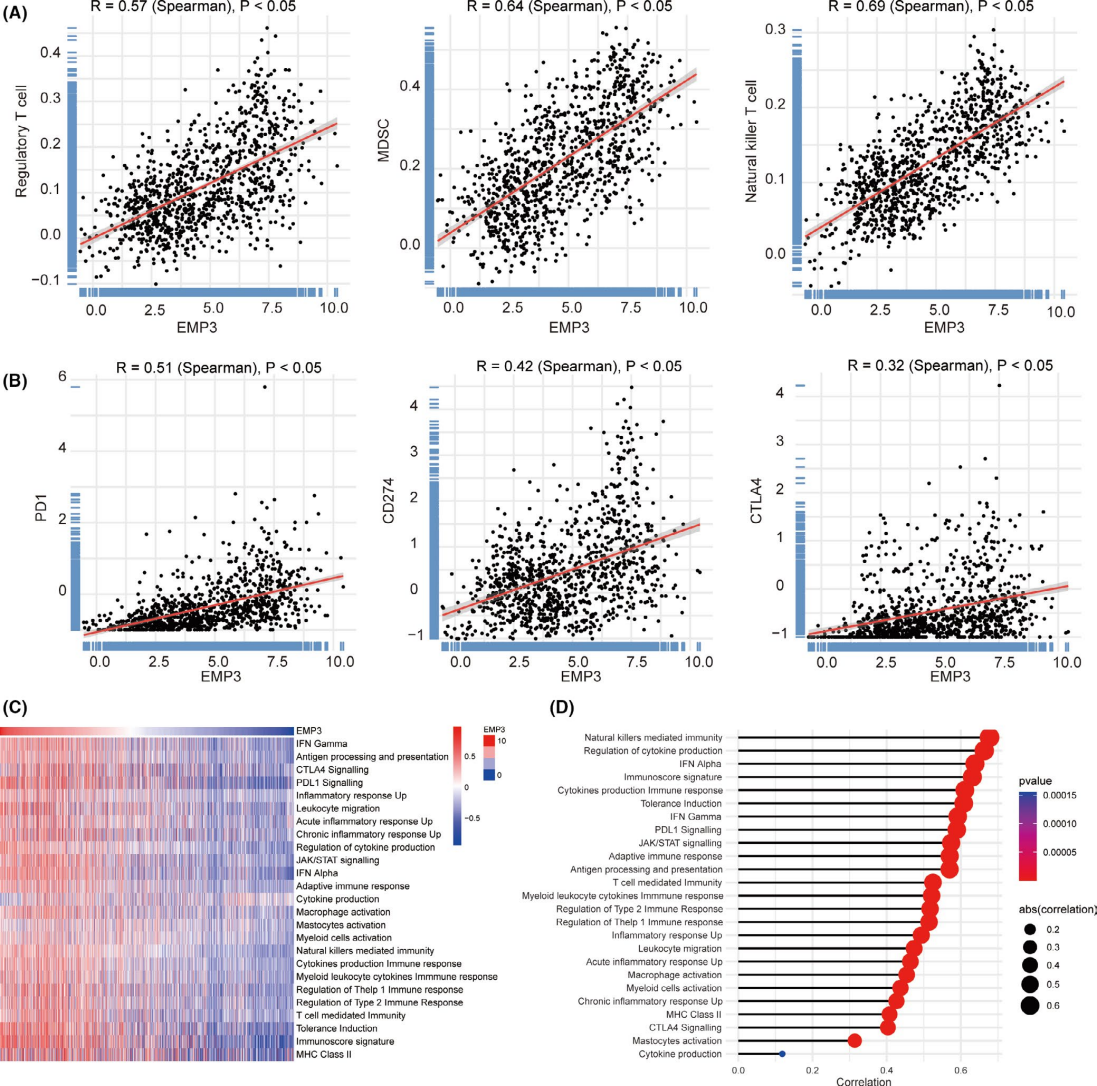
EMP3 is associated with therapeutic targets of PD1/PDL1 and CTLA4 (A) The correlation between the infiltration of regulatory T cell, MDSC, natural killer T cell, and the expression of EMP3. (B) The correlation between EMP3 expression and therapeutic targets of PD1/PDL1 and CTLA4. (C) Heatmap showing EMP3‐associated GSVA scores of 25 innate and adaptive immunity‐related gene sets. (D) The correlation between the GSVA scores of 25 immunity‐related gene sets and the EMP3 levels in gliomas

In our present study, we observed that the majority of immune cells, both of protumor immune cells, including MDSCs and regulatory T cells (Tregs), and antitumor immune cells, such as natural killer (NK) T cells, were significantly increased in high‐grade glioma (Figure 3A). Notably, we observed that patients with high expression of EMP3 also had high expression of the therapeutic targets PD1/PDL1 and CTLA4, which are considered to be immune checkpoint proteins (Figure 3B).

Although the increase in antitumor immune cells in high‐grade glioma seems to be paradoxical, the significant infiltration protumor immune cells might counteract the infiltration of antitumor immune cells and disrupt the balance between the two cell types. To further explore the existence of malignant gliomas with a protumor immune phenotype, manually curated gene sets associated with both adaptive and innate immune responses were used to quantify the immune phenotype (Figure 3C). As is shown in the heatmap, with an increase in EMP3 expression, the tumor microenvironment is more likely to present respond to immune checkpoint blockades (ICBs). This is consistent with the conclusion drawn above that FCER1G plays a critical role in the activation of the immune response. In addition, the results of GSVA revealed a high correlation between the FCER1G and the activated PDL1 pathway (r = 0.57, *p* < 0.05), activated CTLA4 pathway (r = 0.42, *p* < 0.05), and T cell–mediated immunity (r = 0.53, *p* < 0.05) (Figure 3D).

The above findings suggest that EMP3 is involved in immune infiltration remodeling in glioma and is closely associated with T‐cell infiltration, which plays a significant role in immunosurveillance evasion in malignant glioma.^26^

### 3.3 The clinical value of EMP3 for predicting tumor characteristics and survival outcomes

To better understand the clinical role of EMP3 in patients with glioma, we analyzed tumor samples and clinical data from a total of 179 glioma patients. Patients from the Second Hospital affiliated with Zhejiang University were considered the training cohorts, and those from the Shanghai General Hospital were used as the validation cohorts. The demographic features and clinicopathological characteristics of the patients are presented in Table 1. Detailed information on baseline characteristics, tumor malignancy, comprehensive histopathological biomarkers, and EMP3 expression levels is summarized. Although there was a slight difference in Ki67 expression between the two cohorts, there were no differences in the patients’ demographics and the other tumor characteristics.

**TABLE 1:** Clinical characteristics and outcomes of glioma patients from two institutions

	Training cohort (*n* = 91)	Validation cohort (*n *= 88)	*p* value
Gender (Male)	38 (41.8%)	34 (38.6%)	0.785
Age	46.0 ± 15.7	41.9 ± 16.2	0.082
WHO grade			0.318
I	2 (2.2%)	4 (4.5%)	
II	18 (19.8%)	16 (18.2%)	
III	27 (29.7%)	17 (19.3%)	
IV	44 (48.4%)	51 (58.0%)	
Ki67 level			0.034
Low	16 (17.6%)	14 (15.9%)	
Median	22 (24.2%)	9 (10.2%)	
High	53 (58.2%)	44 (50.0%)	
PHH3 level			0.859
Low	46 (50.5%)	44 (50.0%)	
Median	21 (23.1%)	18 (20.5%)	
High	24 (26.4%)	26 (29.5%)	
P53 mutant			0.996
Yes	30 (33.0%)	28 (31.8%)	
No	61 (67.0%)	60 (68.2%)	
IDH1 mutant			0.429
Yes	62 (68.1%)	54 (61.4%)	
No	29 (31.9%)	34 (38.6%)	
ATRX mutant			0.493
Yes	46 (50.5%)	39 (44.3%)	
No	45 (49.5%)	49 (55.7%)	
MGMT methylation			0.120
Yes	57 (62.6%)	44 (50.0%)	
No	34 (37.4%)	44 (50.0%)	
EMP3 expression	13.5 ± 5.6	13.7 ± 6.2	0.853
1‐year follow‐up			0.999
Alive	57 (62.6%)	56 (63.6%)	
Dead	34 (37.4%)	32 (36.4%)	
3‐year follow‐up			0.999
Alive	17 (18.7%)	17 (19.3%)	
Dead	74 (81.3%)	71 (80.7%)	

To validate the clinical role of EMP3, according to the median expression levels of EMP3, we divided the patients from the two centers into a high EMP3 group (*n* = 82) and a low EMP3 group (*n* = 97). By univariate analysis of clinical features, we observed that EMP3 was more likely to be associated with high malignancy (*p* = 0.032), high Ki67 expression (*p* = 0.005), and wild‐type IDH (*p* < 0.001). However, there were no significant differences in age, sex, PHH3 levels, ATRX mutation status, or MGMT methylation levels. In addition, of the patients with tumors with high EMP3 expression, 51.5% and 96.9% had a poor prognosis at 1 year and 3 years after surgery, respectively (Table 2).

**TABLE 2:** Clinical characteristics of 179 glioma patients from two institutions according to EMP3 expression levels

	EMP3 low expression (*n* = 82)	EMP3 high expression (*n* = 97)	*p* value
Gender (Male)	50 (61.0%)	57 (58.8%)	0.764
Age	43.6 ± 15.0	44.7 ± 17.9	0.654
WHO grade			0.032*
I	5 (6.1%)	1 (1.0%)	
II	21 (25.6%)	13 (13.4%)	
III	19 (23.2%)	25 (25.8%)	
IV	37 (45.1%)	58 (59.8%)	
Ki67 level			0.005*
Low	20 (24.4%)	10 (10.3%)	
Median	18 (22.0%)	13 (13.4%)	
High	44 (53.7%)	74 (76.3%)	
PHH3 level			0.213
Low	43 (52.4%)	47 (48.5%)	
Median	21 (25.6%)	18 (18.6%)	
High	18 (22.0%)	32 (33.0%)	
P53 mutant			0.272
Yes	30 (36.6%)	28 (28.9%)	
No	52 (63.4%)	69 (71.1%)	
IDH1 mutant			0.0001*
Yes	42 (51.2%)	74 (76.3%)	
No	40 (48.8%)	23 (23.7%)	
ATRX mutant			0.222
Yes	43 (52.4%)	42 (43.3%)	
No	39 (47.6%)	55 (56.7%)	
MGMT methylation			0.825
Yes	47 (57.3%)	54 (55.7%)	
No	35 (42.7%)	43 (44.3%)	
1‐year follow‐up			0.0001*
Alive	66 (80.5%)	47 (48.5%)	
Dead	16 (19.5%)	50 (51.5%)	
3‐year follow‐up			0.0001*
Alive	31 (37.8%)	3 (3.1%)	
Dead	51 (62.2%)	94 (96.9%)	

The use of * and Bold indicates statistical significance of factors.

In the first year following surgical resection, 66 (36.9%) patients had poor clinical prognosis. In the favorable‐outcome group, there were 66 (58.4%) men and 47 (41.6%) women with a mean age of 43.6 ± 15.0 years, while the poor‐outcome group consisted of 41(62.1%) men and 25 (37.9%) women with a mean age of 44.7 ± 17.9 years. Patients with an unfavorable prognosis tended to have higher WHO grade tumors (*p* = 0.010), higher Ki67 (*p* = 0.049) and PHH3 expression (*p* = 0.023), less IDH1 mutations (*p* = 0.001), and particularly high EMP3 levels (*p* < 0.0001). However, P53 and ATRX mutation status and MGMT methylation levels showed no significant differences. After 3 years, 145 (81.0%) patients had a poor clinical prognosis; among these patients, there were 87 (60.0%) men and 58 (40.0%) women with a mean age of 44.6 ± 16.0 years, and 20 (58.8%) men and 14 (41.2%) women with a mean age of 41.2 ± 16.4 years were still alive. Moreover, WHO grade (*p* < 0.0001), Ki67 expression (*p* < 0.0001), IDH1 mutation status (*p* < 0.0001), and EMP3 levels (*p* < 0.0001) were also associated with poor clinical prognosis (Table 3).

**TABLE 3:** Comparison of Clinical characteristics and comprehensive histopathological biomarkers between favorable and unfavorable outcomes at 1‐year and 3‐year follow‐ups

	1‐year follow‐up			3‐year follow‐up		
	Favorable	Unfavorable	*p* value	Favorable	Unfavorable	*p* value
Number	113 (63.1%)	66 (36.9%)		34 (19.0%)	145 (81.0%)	
Gender (Male)	66 (58.4%)	41 (62.1%)	0.625	20 (58.8%)	87 (60.0%)	0.900
Age	43.6 ± 15.0	44.7 ± 17.9	0.654	41.2 ± 16.4	44.6 ± 16.0	0.268
WHO grade			0.010*			<0.0001*
I	6 (5.3%)	0 (0.0%)		5 (14.7%)	1 (0.7%)	
II	28 (24.8%)	6 (17.6%)		14 (41.2%)	20 (13.8%)	
III	25 (22.1%)	19 (28.8%)		5 (14.7%)	39 (26.9%)	
IV	54 (47.8%)	41 (62.1%)		10 (29.4%)	85 (58.6%)	
Ki67 level			0.049*			<0.0001*
Low	23 (20.4%)	7 (10.6%)		12 (35.3%)	18 (12.4%)	
Median	23 (20.4%)	8 (12.1%)		9 (26.5%)	22 (15.2%)	
High	67 (59.3%)	51 (77.3%)		13 (38.2%)	105 (72.4%)	
PHH3 level			0.023*			0.568
Low	60 (53.1%)	30 (45.5%)		21 (61.8%)	69 (47.6%)	
Median	29 (25.7%)	10 (15.2%)		7 (20.6%)	32 (22.1%)	
High	24 (21.2%)	26 (39.4%)		6 (17.6%)	44 (30.3%)	
P53 mutant			0.147			0.689
Yes	41 (36.3%)	17 (25.8%)		12 (35.3%)	46 (31.7%)	
No	72 (63.7%)	49 (74.2%)		22 (64.7%)	99 (68.3%)	
IDH1 mutant			0.0010*			<0.0001*
Yes	63 (55.8%)	53 (80.3%)		11 (32.4%)	105 (72.4%)	
No	50 (44.2%)	19 (19.7%)		23 (67.6%)	40 (27.6%)	
ATRX mutant			0.838			0.956
Yes	53 (46.9%)	32 (48.5%)		16 (47.1%)	69 (47.6%)	
No	60 (53.1%)	34 (51.5%)		18 (52.9%)	76 (52.4%)	
MGMT methylation			0.185			0.485
Yes	68 (60.2%)	33 (50.0%)		21 (61.8%)	80 (55.2%)	
No	45 (39.8%)	33 (50.0%)		13 (38.2%)	65 (44.8%)	
EMP3 expression	12.0 ± 5.4	16.3 ± 5.5	<0.0001*	7.8 ± 3.9	14.9 ± 5.4	<0.0001*
EMP3 level			<0.0001*			<0.0001*
Low	66 (58.4%)	16 (24.2%)		31 (91.2%)	51 (35.2%)	
High	47 (41.6%)	50 (75.8%)		3 (8.8%)	94 (64.8%)	

The use of * and Bold indicates statistical significance of factors.

### 3.4 Univariate and multivariate analysis of risk factors for overall survival of glioma patients

To determine the clinical predictive value of EMP3 expression, we incorporated factors, including age, sex, WHO grade, expression of Ki67 and PHH3, mutant status of P53, IDH1, and ATRX, methylation level of MGMT, and especially, EMP3 levels of 1013 glioma patients, into the Cox regression model. Combined with univariate analysis and multivariable Cox regression analysis, the association between 3‐year mortality and high malignancy (HR = 2.787, 95% CI: 1.966–3.950), lower IDH1 mutation rates (HR = 0.503, 95% CI: 0.344–0.773), and high EMP3 levels (HR = 2.842, 95% CI: 1.984–4.071) remained significant in the forward stepwise multivariable model. Although the univariate analysis suggested that high expression of Ki67 and PHH3 were predictors for 3‐year poor outcome, they were not predictors in the final model. The expression level of EMP3 in glioma patients was significantly correlated with the OS rate. The expression level of EMP3 is a stabilizing factor affecting the survival time of glioma patients (Table 4).

**TABLE 4:** Univariate and multivariate Cox regression analysis for overall survival of glioma patients

	Univariate analysis		Multivariate analysis	
	HR (95% CI)	*p* value	HR (95% CI)	*p* value
Age (≥50 vs <50)	1.192 (0.853–1.668)	0.283		
Gender (Male vs Female)	1.079 (0.776–1.501)	0.462		
WHO grade (HGG vs LGG)	2.787 (1.966–3.950)	**<0.001** ^a^	1.991 (1.235–3.212)	**0.005** ^a^
Ki67 expression (High vs Low)	1.869 (1.343–2.600)	**<0.001** ^a^		
PHH3 expression (High vs Low)	1.505 (1.019–2.223)	**0.019** ^a^		
P53 status (Mutant vs WT)	1.127 (0.7997–1.587)	0.494		
IDH1 status (Mutant vs WT)	0.395 (0.285–0.547)	**<0.001** ^a^	0.503 (0.344–0.737)	**<0.001** ^a^
ATRX status (Mutant vs WT)	0.973 (0.703–1.349)	0.868		
MGMT methylation (Yes vs No)	1.118 (0.804–1.555)	0.493		
EMP3 expression (High vs Low)	3.029 (2.166–4.328)	**<0.001** ^a^	2.842 (1.984–4.071)	**<0.001** ^a^

Note

The use of * and Bold indicates statistical significance of factors.

Factors with *p* ≤ 0.05 in univariate analysis can be included in the multivariate analysis.

Abbreviations: CI, confidence interval; HR, hazard ratio.

^a^ *p* ≤ 0.05.

### 3.5 Establishment of nomogram and validation of predictive accuracy for poor outcome

**FIGURE 4:**
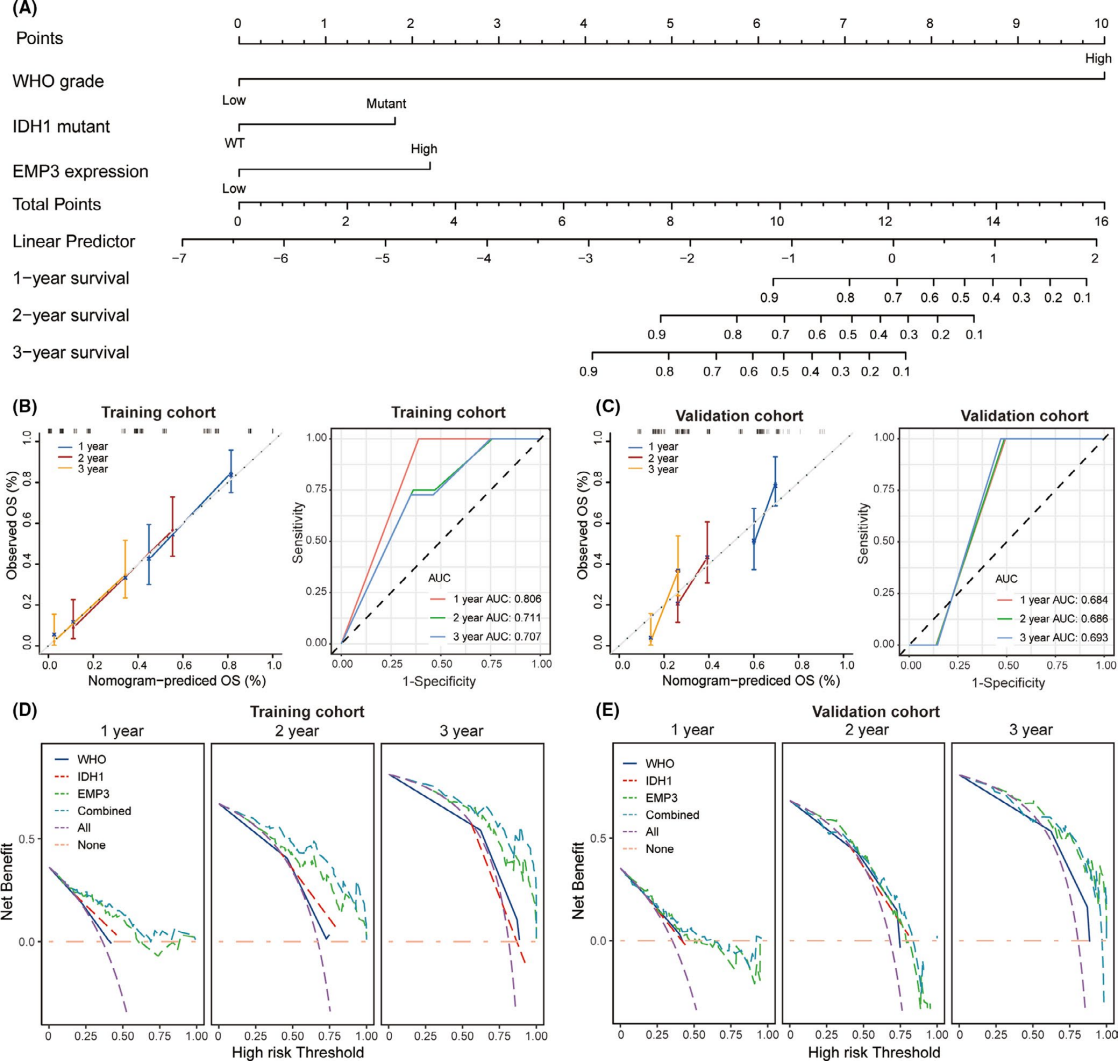
Nomogram for clinical outcome at 1‐, 2‐, and 3‐year follow‐up. (A) To evaluate the probability of disability for an individual patient, review his/her clinical data and image features list in nomogram. Then, draw a vertical line from the feature status toward the Points axis to obtain respective points based on each feature. Finally, draw a vertical line through the Total points axis, according to the sum of the total score, which will intersect the probability of poor outcomes axis at the predicted probability. (B) Calibration curve and time ROC curve for nomogram in training cohort. The gray line represents performance of ideal nomogram where the predicted probability perfectly corresponds with observed probability. (C) Calibration curve and time ROC curve for nomogram in training cohort. (D and E) DCA curve to verify the prognostic performance of the model by comparison with single factors in training cohort (D) and validation cohort (E)

To better apply the results of this study to clinical practice, we established a predictive nomogram based on the multivariable regression analysis to predict the 1‐, 2‐, and 3‐year prognosis of patients (Figure 4A). This nomogram showed the risk stratification with the factors of WHO grades, IDH1 mutation status, and EMP3 expression. For an individual patient, risk factors contributed to respective points and the sum corresponded to the probability of 1‐, 2‐, and 3‐year mortality. Then, to confirm the practical value of this model, we performed a calibration plot. The calibration curves revealed that the nomogram was relatively well calibrated and corresponded with prediction and observation. In addition, in the validation cohort, the calibration curve presented minor discrepancies between observed and predicted probabilities (Figure 4B and C). As shown in Figure 4, the AUCs of the 1‐, 2‐, and 3‐year nomograms of the training cohort were 0.806, 0.771, and 0.707, respectively, while the AUCs of the validation cohort were 0.684, 0.686, and 0.693, respectively.

Then, we analyzed these three independent predictors using decline curve analysis (DCA) to confirm the predictive ability of the nomogram for the 1‐, 2‐, and 3‐year overall survival rates of the patients. The curves demonstrated that the nomogram with combined factors was significantly better than individual factors alone in prognostic prediction. In addition, high expressed EMP3 is of great predictive value and accuracy in prognostic prediction of glioma patients (Figure 4D). To demonstrate the generality of the nomogram model, a validation cohort set was used to further confirm the prediction value of the model (Figure 4E). According to the above analysis, the establishment of this model is undoubtedly of substantial significance to both clinician workers and patients.

## 4 DISCUSSION

To the best of our knowledge, there are few studies applying human glioma samples to verify signatures or biomarkers screened from public datasets. Recent studies have identified various molecules for both therapeutic targets and prognostic predictors in gliomas, including RGS16, NLR, TMEM7, SLAMF8, LINC00152, and NUSAP1.^27^, ^28^, ^29^, ^30^, ^31^, ^32^, ^33^ Above novel prognostic factors tend to facilitate tumor progression *per se*; however, the protumor and immunomodulatory effects of EMP3 were both demonstrated previously.^34^ Due to rare studies clarified the biological function and clinical value of EMP3 in glioma, here, we combined bioinformatic analysis and clinical data and established a nomogram predictive model with bicenter validation.

The relationship between EMP3 expression and tumors has been revealed in a series of previous studies, with controversial results regarding its role as an oncogene or tumor suppressor in many solid cancers. In upper urinary tract urothelial carcinoma, EMP3 enhances tumor progression the ErbB2‐PI3K‐AKT signaling pathway.^18^ In addition, EMP3 can promote hepatocellular carcinoma (HCC) by activating the PI3K/AKT pathway and uPA/MMP‐9 cascade, upregulation of which is closely associated with differentiation.^17^ However, in comparison, EMP3 might act as a tumor suppressor in esophageal carcinoma and lung cancer.^20^, ^21^ The above findings suggested that the function of EMP3 in solid tumors might be multifaceted and dependent on the specific type of cancer.

Although EMP3 was initially identified as a tumor suppressor in low‐grade gliomas, its inhibitory role is still controversial. EMP3 expression was significantly higher in GBM than in non‐neoplastic white matter and led to worse OS rates in WHO grade II‐III glioma.^23^, ^35^ Another recent study reported that EMP3 directly interacts with TGFBR2 in glioma cells, which subsequently activates the TGF‐β/Smad2/3 pathway and enhances tumor progression in vitro and in vivo.^22^ In our present study, we determined that EMP3 enhanced glioma progression and showed clinical value for prognostic prediction.

Tumor initiation and progression is a complex process that requires interactions among cancer cells, the tumor microenvironment, and the immune system.^36^ Recent studies have revealed that immune cells within the tumor microenvironment have an accessory role in maintaining tumor homeostasis and can influence the clinical outcome of tumors.^37^, ^38^ In our previous study, we revealed that FCER1G is a novel predictor for clinical prognosis and response to immunotherapy in glioma patients, which is of great clinical significance and will contribute to the development of individualized management plans.^39^

EMP3 expressed by antigen‐presenting cells (APCs) induces alloreactive cytotoxic T lymphocytes (CTLs) via TNF‐α production. The clarification of the role of EMP3 may lead to a cure for malignant tumors.^40^ In this study, we revealed that there was more immune cell infiltration in EMP3^high^ glioma. In addition, we observed that the levels of the majority of immune cells, both protumor immune cells, including Tregs and MDSCs,^41^ and antitumor immune cells ^42^ were significantly increased in high‐grade glioma. Although the increase in antitumor immune cells in high‐grade glioma seems to be paradoxical, the significant infiltration of protumor immune cells might counteract the infiltration of antitumor immune cells and disrupt the balance between the two cell types. Notably, we observed that patients with high EMP3 expression also had high expression of the immune checkpoints PD1/PDL1 and CTLA4, which may explain why immune activation was enriched in the EMP3 high subgroup, but did not hinder tumor progression.

In addition to EMP3, WHO grading and IDH1 mutation status were also included in this practical nomogram. Although the mutation of isocitrate dehydrogenase enzyme (IDH) has been proven to be an inciting event in glioma‐genesis, recent genome‐wide mutation analyses have revealed the prevalence of IDH mutations in more than 70% of WHO grade II and III gliomas or secondary GBMs, whereas fewer than 5% of primary GBMs harbor this mutation.^43^ In addition, a variety of studies have suggested that IDH mutation is related to better outcome and sensitivity to chemotherapy.^44^, ^45^ According to various clinical studies, glioma patients with IDH mutations show longer OS and progression‐free survival rates than their counterparts with IDH wild‐type IDH.^46^, ^47^, ^48^ Translating the results of these studies into clinical practice is the ultimate goal. One of the highlights of this predictive model is the combination of clinical characteristics and biomarkers, making this model more clinically practical.

There are several limitations in our study. First, the patient outcome data of patients were recorded from outpatient and telephone interviews at 1, 2, and 3 years, for which there was referral bias due to unacceptable and incoordinate patients with unfavorable neurological status or clinical outcomes. Thus, a potential underestimation of poor prognosis might have influenced the overall estimate. Additionally, because of the relatively limited number of patients, there is potential for minor bias to skew the interpretation.

## 5 CONCLUSION

This study demonstrated that EMP3 is a novel independent predictor for clinical diagnosis, prognosis, and immune infiltration in glioma patients. These results are of great clinical significance and will contribute to prognostic prediction and the development of individualized therapies. Although more clinical data from other institutions are required for further validation of our nomograms, individualized quantitative risk assessment using the present nomograms would be a practical approach for predicting prognosis and counseling patients.

## CONFLICT OF INTEREST

The authors declare that the research was conducted in the absence of any commercial or financial relationships that could be constructed as a potential conflict of interest.

## AUTHOR CONTRIBUTIONS

JZ and ML: conception, supervision, and design of this article. AZ and ZZ: data analysis and editing the manuscript. XH, YL, LY, and YN: data collection and patients’ follow‐up. SG, SC, JJ, and YX: tumor sample collection and IHC analysis. HX, YC, and XZ: bioinformatic analysis. All authors contributed to the article and approved the submitted version.

## Supporting information

Figure S1

## Data Availability

Publicly available datasets were analyzed in this study. These data can be found here: http://gliovis.bioinfo.cnio.es/. The supplementary material for this article can be found online. All processed data and R codes used in this study can be obtained from the corresponding author on reasonable request.
