# Supplementary figures and images for: Establishment of a nomogram with EMP3 for predicting clinical outcomes in patients with glioma: A bi‐center study
Source: CNS Neurosci Ther. 2021 Jul 16;27(10):1238–50. doi: 10.1111/cns.13701 (PMC8446216; doi:10.1111/cns.13701)

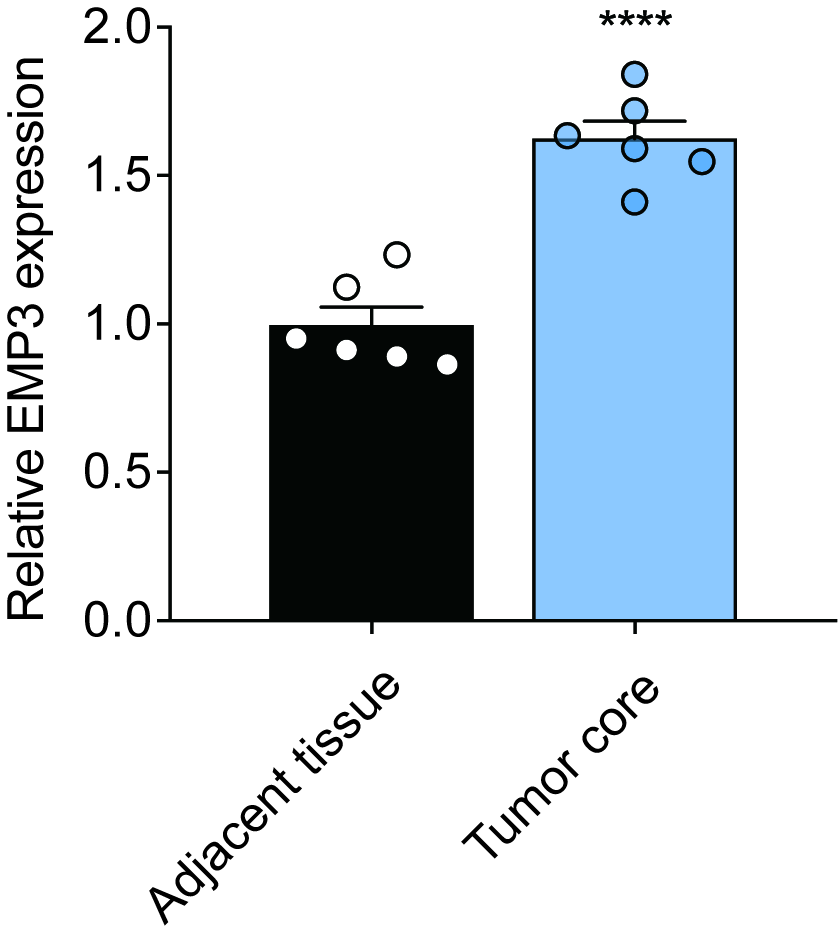

Supplement: Supplementary file 1 — Figure S1 [file CNS-27-1238-s001.tif]
